# The Feasibility and Acceptability to Service Users of CIRCuiTS, a Computerized Cognitive Remediation Therapy Programme for Schizophrenia

**DOI:** 10.1017/S1352465815000168

**Published:** 2015-05-25

**Authors:** Clare Reeder, Victoria Pile, Polly Crawford, Matteo Cella, Diana Rose, Til Wykes, Andrew Watson, Vyv Huddy, Felicity Callard

**Affiliations:** Institute of Psychiatry, Psychology and Neuroscience, King's College London, UK; University College London, UK; University of Durham, UK

**Keywords:** Schizophrenia, cognitive remediation, feasibility, acceptability, service user, cognition

## Abstract

**Background:** Cognitive remediation (CR) is a psychological therapy, effective in improving cognitive performance and functioning in people with schizophrenia. As the therapy becomes more widely implemented within mental health services its longevity and uptake is likely to depend on its feasibility and acceptability to service users and clinicians. **Aims:** To assess the feasibility and acceptability of a new strategy-based computerized CR programme (CIRCuiTS) for people with psychosis. **Method:** Four studies were conducted using mixed methods. Perceptions of attractiveness, comprehensibility, acceptability and usability were assessed using self-report questionnaires in 34 non-clinical participants (study 1), and five people with a schizophrenia diagnosis and three experienced CR therapists (studies 2 and 3). The ease with which pre-specified therapy programmes could be assembled was also assessed by three therapists (Study 2). Finally, the satisfaction of 20 service users with a diagnosis of schizophrenia regarding their experience of using CIRCuiTS in the context of a course of the CR therapy was assessed in a qualitative interview study (study 4). **Results:** Ratings of perceived attractiveness, comprehensibility, acceptability and usability consistently exceeded pre-set high targets by non-clinical, clinical and therapist participants. Qualitative analysis of satisfaction with CIRCuiTS showed that receiving the therapy was generally seen to be a positive experience, leading to perceptions that cognitive functioning had improved and attempts to incorporate new strategy use into daily activities. **Conclusions:** CIRCuiTS demonstrates high acceptability and ease of use for both service users with a schizophrenia diagnosis and clinicians.

## Introduction

Cognitive Remediation (CR) is a psychological therapy to improve thinking skills, and is effective in improving cognitive performance and functional outcomes in people with a diagnosis of schizophrenia (Wykes, Huddy, Cellard, McGurk and Czobor, 2011). Consequently it is beginning to be recommended by governmental guidelines (e.g. SIGN, 2013) and the field is moving towards widespread implementation and identifying critical factors for an effective and easy-to-implement therapy (Wykes and Spaulding, 2011).

CR programs are largely based on the core principles of massed practice, error-less learning and self-motivation, but procedural and implementation differences are common, in terms of method of delivery, design interface, reliance on a therapist, and the frequency and length of sessions. Furthermore, CR programmes differ in the extent of their focus on the transfer of cognitive skills to general functioning, but generally, approaches incorporating elements of strategy-development as well as adjunctive rehabilitation have been more effective for functional improvements (Wykes et al., 2011).

Computerized delivery may be particularly attractive as there is growing consensus that intensive and prolonged engagement is required (Keefe et al., 2011), and access to psychological therapists is scarce. Pragmatic factors such as these are likely to play an important part in driving the selection of a CR programme for health service implementation. Most crucially, the uptake and durability is likely to be influenced by its acceptability to users (e.g. Drake, Csipke and Wykes, 2013). However, most CR programmes have been designed for no specific diagnostic group, or with little user consultation. This may be particularly important as evidence emerges to emphasize the pivotal role of intrinsic motivation in promoting positive outcomes following CR (Medalia and Richardson, 2005). It is also reflected in a more general consensus that a key priority in the development of treatment programmes and research is service user involvement (e.g. Department of Health, 2000). A recent systematic review of 12 studies of the acceptability and feasibility of internet and mobile-based interventions for people with psychosis included no studies of CR, and concluded that although most programmes were used effectively and perceived as positive and useful, relevant research is currently in its early stages (Alvarez-Jiminez et al., 2014).

Our team developed a new CR programme for people with a schizophrenia diagnosis, namely CIRCuiTS (Computerized Interactive Remediation of Cognition – a Training for Schizophrenia; Reeder and Wykes, 2010) to bring together (a) existing knowledge regarding best practice to promote cognitive and functional change; and (b) feasibility of implementation. It is ambitious in fostering the transfer of new cognitive skills to everyday life within the programme itself, rather than relying on bridging sessions or adjunctive rehabilitation. This is underpinned by a model that suggests that cognitive skills will only be used to benefit everyday functioning if (a) metacognitive knowledge about (i) one's own strengths and difficulties, and (ii) how thinking skills and strategies can impact behaviour in general, and (b) metacognitive regulation of one's own behaviour (cf executive functioning) are both used to guide the adoption of new behaviours (Wykes and Reeder, 2005). This model is gaining initial support from evidence that executive function changes are better predictors of functional change in schizophrenia than change in basic cognitive processes (e.g. Reeder et al., 2014; Reeder, Newton, Frangou and Wykes, 2004; Reeder, Smedley, Butt, Bogner and Wykes, 2006).

CIRCuiTS has a strong emphasis on individual tailoring. It is a modular system that is adaptable to: (i) different cognitive problems, as therapists can create therapy programmes composed of any combination of differently focused cognitive tasks; (ii) different cognitive abilities, through task selection and artificial intelligence systems that adjust task difficulty; (iii) different languages; (iv) cultural differences through ready accessibility to content changes (Press, Drake and Husain, 2010), and (v) settings, available in both online and offline versions and for therapist-led sessions and independent homework sessions. Second, its development has been based on consultation with service users with a schizophrenia diagnosis. It is this process that is reported in this paper.

Service user involvement relies on the premise that involving patients directly in service or research development will lead to more accessible and acceptable services or studies (Barker, Bullen and de Ville, 1997), by eliciting unique viewpoints and specific expertise by experience (Ennis and Wykes, 2013). There has been a growing emphasis on ensuring very early service user involvement in translational research to optimize the chance that outputs – such as a computerized therapy programme – will be appropriately designed for their end users (Callard, Rose and Wykes, 2012).

## Aims

We report four studies, using mixed methods, to ensure maximum usability and acceptability to users of CIRCuiTS. The general public, service users with a schizophrenia diagnosis and therapists took part. In particular, we were interested in ensuring that all groups found the programme (a) attractive, (b) culturally acceptable, (c) easy to understand and (d) easy to use.

These studies formed part of a comprehensive, development programme in which we took an initial version 1 (v1) prototype of CIRCuiTS, which had already undergone a first stage of feasibility testing, through an iterative development process, to a final completed version (v2.1), which was then tested in a feasibility randomized controlled trial. The aims of the four studies were:

1. To investigate perceptions of attractiveness, comprehensibility, acceptability and usability of CIRCuiTS v1 and v2 within:
  a a non-clinical sample
  b a sample of people with a schizophrenia diagnosis
  c experienced CR therapists.
2. To assess the ease with which pre-specified therapy programmes can be assembled by therapists.
3. To investigate qualitatively the satisfaction of service users with a diagnosis of schizophrenia regarding their experience of using CIRCuiTS in the context of a course of the CR therapy.

CIRCuiTS has already been subject to independent investigation in a naturalistic randomized controlled trial (RCT) in which it was combined with cognitive behaviour therapy for psychosis (CBTp) in a first episode sample (Drake et al., 2014). CBTp courses preceded by CIRCuiTS were significantly shorter but achieved the same outcome as CBTp preceded by an active control.

### Description of CIRCuiTS

CIRCuiTS is a web-based computerized CR programme, with alternative offline installations, delivered primarily by a therapist but also carried out independently. It is based around a virtual “village” and the tasks take place in relevant buildings. There are 27 tasks, each with at least 12 difficulty levels, which are partially regulated by artificial intelligence.

**Figure 1.**
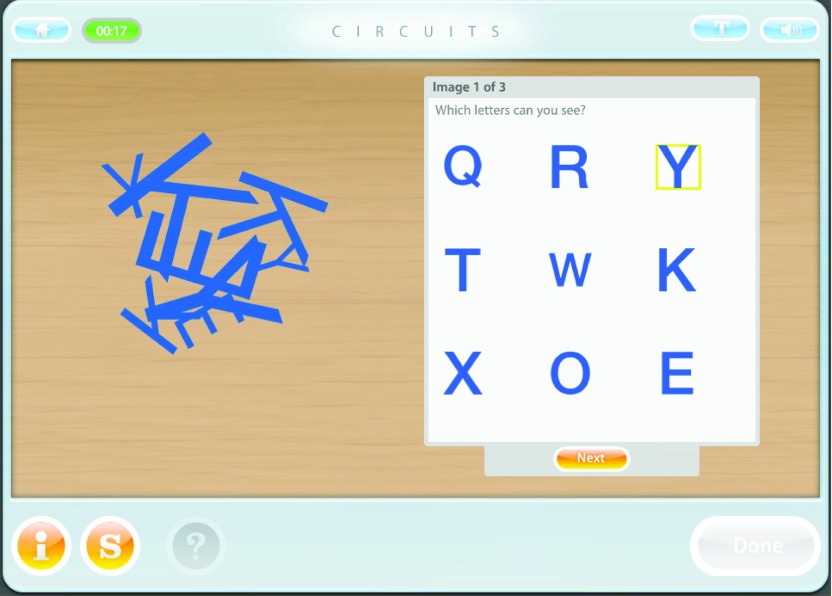
CIRCuiTS abstract task

*A focus on transfer within the programme*. Tasks are divided into two types: “abstract tasks”, and “exercises”. Abstract tasks have neutral content (e.g. number or geometrical shapes) and are designed to target specific cognitive functions (see Figure 1). They are predominant in the early phase of the programme and gradually become more interspersed.

**Figure 2.**
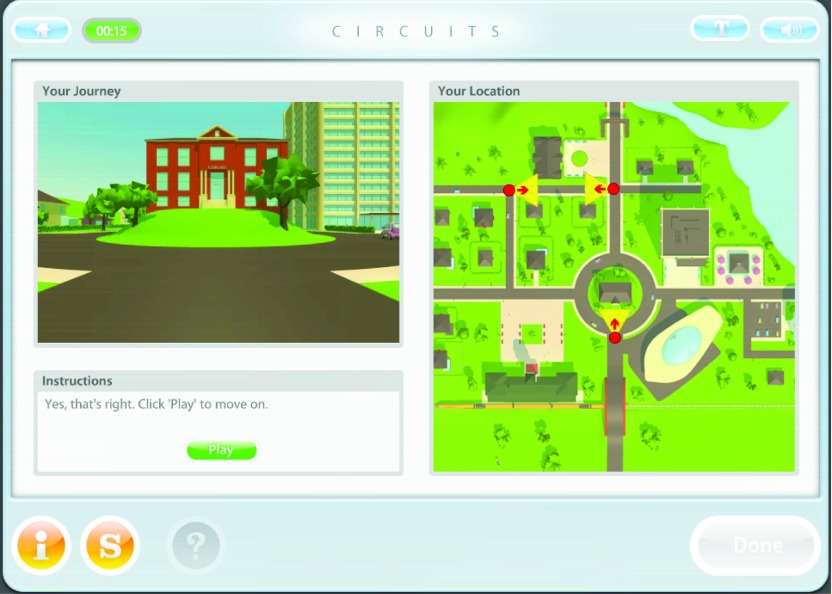
CIRCuiTS exercise

Exercises are more complex, ecologically-valid tasks, which require multiple cognitive functions, particularly executive functions (see Figure 2). They fall within five categories: work, social situations, cooking, shopping, and travelling. They are gradually introduced throughout the programme. This is one of the ways in which generalization is specifically targeted within the CR programme itself. In addition, the programme and therapist make explicit attempts to encourage the service user to draw parallels between within-session tasks and everyday life and to rehearse new skills in vivo.

*Metacognitive model implementation into the programme*. The development of metacognition is explicitly targeted through a “metacognitive journey”: (1) introduction to CIRCuiTS; (2) your goals; (3) your strategies; (4) your daily life; and (5) your achievements. Service users set goals and identify personal strengths and difficulties, which are regularly reviewed and modified.

Learning supports accompany each task to encourage metacognition. Before each task, service users (a) rate the anticipated difficulty of the task; (b) estimate how long the task will take; and (c) identify strategies to complete the task. After the task, they see their score and rate (a) how difficult they actually found the task and (b) how useful they found their strategies.

The therapist also focuses on metacognition: the service user is encouraged to adopt a systematic approach: planning, implementation and review. They also work collaboratively to gain an understanding of the service user's own cognitive and non-cognitive (e.g. mood, sleep, medication effects, beliefs, environment) strengths and difficulties that affect performance. Based on this shared understanding, the service user develops a personalized flexible set of strategies.

*Individually tailored treatment:* An administrator interface allows therapists to manage and create new therapy programmes comprising any combination of CIRCuiTS tasks and metacognitive elements to address individual patient needs.

### CIRCuiTS development programme

**Figure 3.**
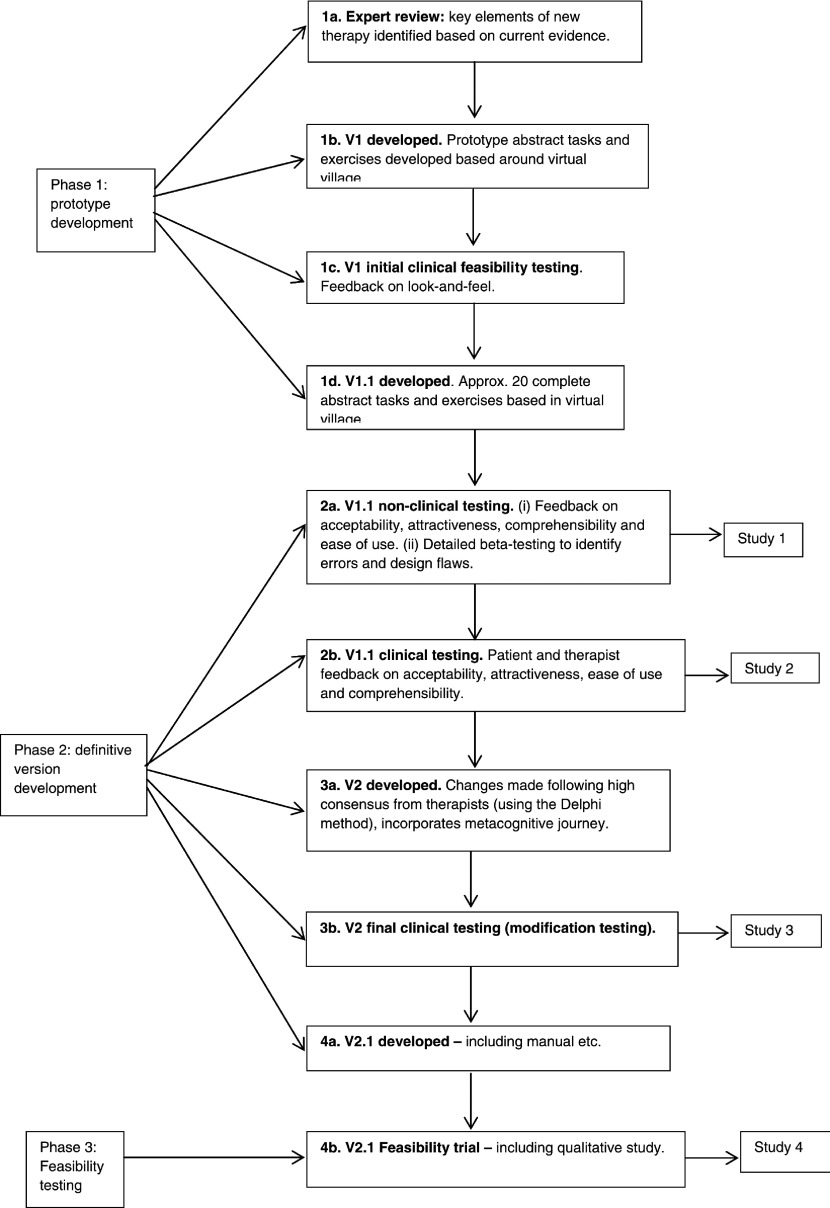
CIRCuiTS development process

CIRCuiTS was developed within a comprehensive iterative programme (see Figure 3), designed in consultation with CR experts (both clinicians and researchers) and service users with a schizophrenia diagnosis.

## Method: the studies

Ethical permission was granted by King's College London (reference number PNM/08/09-125) (studies 1-3) and the Joint South London and Maudsley and the Institute of Psychiatry NHS Research Ethics Committees (reference number 08/H0807/26) (study 4).

### Study 1: non-clinical testing

*Participants:* Thirty-four non-clinical participants (18–65 years), proficient in English, were recruited through advertisements on a healthy volunteer database and at the local government employment centre. Exclusion criteria: (1) history of psychiatric illness (K6 self-report screening measure, Furukawa, Kessler, Slade and Andrews (2003); (2) history of head injury or other neurological illness or endocrine disorder affecting brain function; (3) current drug or alcohol problems. All participants gave written informed consent and were paid. Demographic data are shown in Table 1.

**Table 1.** Demographic data

	Study 1: Non-clinical	Study 4: Clinical
	participants	participants
Demographic	Percentage / median range	Percentage / mean
Age	26-35 yrs	Mean 39.05 (*SD* 10.29)
Gender	50% men	80% men
Ethnicity	73.5% white 8.8% black 11.8% Asian 5.9% mixed race	20% white 80% ethnic minority
Marital status	47.1% married 47.1% single 5.9% separated or divorced	Not collected
Time since first contact with psychiatric services	N/A	5%: Up to 1 year 15%: 1-5 years 10%: 6-10 years 70%: More than 10 years
Education	88.1% graduates 9.0% left school at 18 years 2.9% left school at 16 years	Mean no. of years 13.03 (*SD* 2.01)
Employment	55.9% full-time or part-time employed 23.5% students 8.8% retired 11.8% unemployed	5% employed 10% unpaid employed 15% students 70% unemployed
Computer use	91.2% 5+ years computer use experience 8.8% 1-5 years computer use experience 26.5% used computer once a day 73.5% used computer throughout the day	Not collected

*Measures:* Measures used are described in Table 2.

**Table 2.** Measures for studies 1 and 2

Measure	Description
Study 1
Self-Assessment Measure (SAM) **(Appendix 1)**	A self-report questionnaire developed in conjunction with service users. Using a 5-point Likert scale, participants rate: (a) attractiveness; (b) ease of understanding and use; and (c) culturally acceptability of specific aspects of the programme as well as CIRCuiTS as a whole
Study 2
Self-Assessment Measure 1 (SAM1)	Adapted to refer to the experience of a completed CIRCuiTS session
Therapist Assessment Measure (TAM) **(Appendix 2)**	Similar to the SAM1, but referred to the therapists' experience of accessing, interpreting and applying all elements of the help functions for both patients and therapists
Therapist Semi-Structured Interview (TSSI) **(Appendix 3)**	Included 5 questions and prompts to assess (a) therapists' perceptions of the administrator interface, specifically the facilities that allow therapists to create new therapy programmes; and (b) their views about CIRCuiTS in general. Responses were recorded and transcribed.

*Procedure:* Participants carried out a series of CIRCuiTSv1.1 tasks and then completed the Self-Assessment Measure (SAM). All CIRCuiTS tasks were assessed by at least one participant.

*Performance: targets, achievement and software changes*. Minimum performance targets on the SAM were pre-specified by the research team and approved by the funding body prior to the start of the study. Failure to achieve the minimum target prevented continuation to service user testing for the overall ratings, or led to appropriate specific amendments to CIRCuiTS for the specific item ratings. Target scores as well as actual performance ratings are shown in Table 3.

**Table 3.** Studies 1-3 performance targets and achievement

	Minimum target rating	Study 1 SAM mean actual ratings (range for specific ratings)	Study 2 SAM1 mean actual ratings (range for specific ratings)		Study 2 TAM mean actual ratings (range for specific ratings)	Study 3 SAM1 actual ratings	Study 3 TAM actual ratings
			After session 1	After session 10			
Attractiveness: Overall rating Specific item ratings	70% 70%	84% 80.8% (67.5% - 85.5%)	88.0% 79.0% (64-84%)	92.0% 92.3% (88-96%)	N/A	76.0% 78.8% (68-88%)	93.3% 81.9% (60-87%)
Comprehensibility and ease of use: Overall rating Specific item ratings	60-70% 70%	77.6% 81.7% (75.9% - 89.4%)	70.0% 71.8% (64-76%)	90.0% 86.5% (75-96%)	N/A	86.0% 82.2% (72-92%)	N/A
Cultural acceptability	</ = 1 difficulty	89%; no difficulties identified	92%; no difficulties identified	96%; no difficulties identified	N/A	80%; no difficulties identified	N/A
Understanding of strategy selection and use, and online help	50-70% (90% study 3)	N/A	70%	85%	N/A	79.0%	95.0%
Ease of understanding: Overall rating Specific item ratings	80% 80%	N/A	N/A	87.0% 85.6% (66.7–93.4%)	N/A	90.0% 85.0% (60-90%)
Ease of use: Overall rating Specific item ratings	80% 80%	N/A	N/A	86.7% 91.1% (86.7%-100%)	N/A	100.0% 95% (90-100%)
	Maximum target time			Study 2 mean actual time taken		
Therapy programme building	20 minutes each	N/A	N/A	12 minutes, 6 seconds All programmes built in <20 minutes	N/A	N/A
TSSI responses:
1. Building therapy programmes 2. Improving CIRCuiTS in general	1. Two therapists described the function as easy to use and useful. The third said it was “laborious” and that they would be more likely to stick to pre-defined programmes 2. Specific suggestions related to the sound and the demos (one therapist) (already identified in TAM ratings), and having a range of pre-defined therapy programmes (one therapist)

Only the specific rating of the attractiveness of the music fell below the performance target. To address this, the CIRCuiTS music was uncoupled from auditory feedback so that users were able to switch off the music without affecting the tasks.

#### Study 2: service user testing

*Participants*. Five people with a clinical diagnosis of schizophrenia (three men; aged 41–56 years; two white British, two black African and one black British); and three experienced CR therapists working within local National Health Services, were recruited.

*Measures*. See Table 2.

*Procedure*. The five service users each received 10 hours of individual CIRCuiTSv1.1 therapy (i.e. 10 sessions). Each person's programme was different and all CIRCuiTSv1.1 content was viewed by at least one participant. At the end of each session, the participant completed the Self-Assessment Measure 1 (SAM1). They were paid for their participation.

The three therapists completed the Therapist Assessment Measure (TAM) following a 1-hour demonstration of CIRCuiTSv1.1. They then assembled five pre-specified therapy programmes to test ease of use following a brief training (10 minutes), and then completed the Therapist Semi-Structured Interview (TSSI). The target time for assembling each therapy programme was 20 minutes.

*Performance: targets, achievement and software changes*. As before, performance targets were pre-specified and minimum target decisions approved. In addition to general targets for SAM1 items, specific target items referred to the ability to understand the learning supports including strategy selection and use, and the online help. These results are all shown in Table 3.

For the TAM, only the specific rating of understanding of the demonstration tasks fell below the performance target. To address this, new demonstration versions of every task were built addressing the specific concerns of therapists (that the speed of demonstration was too rapid).

### Study 3: modification testing

*Participants:* Five people with a diagnosis of schizophrenia (three men; aged 26–55; one white British, one Chinese, one Caribbean British; demographics of two not recorded) and two therapists completed one session each of CIRCuiTS v2.1, with a CRT therapist, and then completed the SAM1 or TAM. The service users were paid for their time.

*Performance: targets, achievement and software changes*. The results of these assessments are shown in Table 3. Pre-specified performance SAM1 and TAM targets were met except for music, which had not been changed but could now be switched off.

### Study 4: qualitative assessment of the experience of using CIRCuiTS

*Participants*. Participants had all received CIRCuiTSv2.1 as part of an RCT comparing CIRCuiTS plus treatment-as-usual (TAU) with TAU alone. CIRCuiTS was delivered several times a week for up to 12 weeks for 20-40 sessions. Inclusion criteria: a DSM-IV diagnosis of schizophrenia; at least 1 year contact with mental health services; aged 17 to 65 years; inefficient executive/memory performance; poor social functioning. Exclusion criteria: a plan to change medication; diagnosis of substance dependence; evidence of organic cause to cognitive difficulties.

Twenty-three participants were contacted and 20 consented to be interviewed. Sampling was by convenience, but these were amongst the first people to complete participation in the RCT. Eighteen of the consented individuals had completed the therapy and two dropped out after only one session (one due to therapy being too time-consuming, the other due to perceived lack of cognitive difficulties). All but one participant was taking atypical anti-psychotic medication.

*Procedure*. Individual semi-structured interviews were carried out by a service-user researcher independent of the trial team. The interview topic guide was developed as an adaptation of the service-user reference group driven satisfaction questionnaire, used in an evaluation of a paper and pencil version of CR (Rose et al., 2008). Other questions were added to incorporate aspects specific to CIRCuiTS (such as computer use). Interview topics included: (i) computer use; (ii) interaction with the therapist; (iii) acceptability of CIRCuiTS tasks; (iv) perceived cognitive changes; (v) general well-being; (vi) positive and negative aspects of the therapy; and (vii) ending the therapy.

All participants gave written informed consent. Interviews were recorded and transcribed. Basic demographic and clinical information at the time of randomization was collected from the RCT researchers.

### Data analysis

Open question answers underwent a simple thematic analysis to identify common experiences in using CIRCuiTS, using an iterative process to extract themes, by a service user researcher, checked by a second senior service user researcher. Consensus on the themes was finalized by discussion. Quotations are included as examples of representative views (see Table 4).

**Table 4.** Service user quotations by theme

Theme	Quotations
Computer use	“It was the actual computer what I got to understand the best and I think I still can remember how to use it.” “I did (like it) because initially I haven't got much knowledge about computers, so at first I thought it was going to be difficult but it was simple. It wasn't anything difficult.”
Design	“I found it quite vibrant and quite easy to understand relatively, yeah, and it's certainly attention catch, it catches your attention, keeps you focused on it I think. . .”
Tasks	“. . . quite challenging some of them, they, they caught me off guard a lot of the time and I'd like to know if I could get better at that. But I found that quite fun.”
Perceived cognitive changes	“It was quite good, very useful for my memory” “I didn't expect the computer programme, . . . to help me with my general well-being and general planning, . . . so I was impressed by it because I didn't think it would work as much as it did actually, so therefore I have to say that I'm impressed by it, yeah.”
Transfer: real life application of CR principles	“. . .after the sessions I became more aware of myself, my weakness and I tried to overcome that, so since then, I've not had that problem. I don't miss anything anymore.” “Yesterday I had to buy four or five things for my mum, I said them in my head over and over again for about 15 seconds, I said it out loud and so I remembered it just came back to me. . .” “As soon as I left, yeah, I tried to remember the stuff I had to remember for the next day. I used to sit on the train and it could stay in my head for 2 or 3 minutes and the next day it helped me more to remember.”
The therapist	“There was a trust, a feeling of trust that I had and a feeling that I was sort of you know in a comfortable environment and in, in kind of like you know everything was being handled in a thoroughly professional manner.” “I liked them because they gave me confidence in myself. . .”
Endings	“. . .I walked away feeling quite sort of, feeling quite challenged and feeling quite maybe refreshed. It was quite intellectually stimulating and I felt quite sort of, in, in a fairly sort of let me see I think contemplative mood.” “I felt a bit sad that it'd come to an end but I felt good that I'd finished it.”

## Results

### Emerging themes

#### Computer use

All but two participants rated this positively. Eight said that they felt they had learnt a new skill. Reasons for liking the computer differed, e.g. (a) already being familiar with using a computer; (b) computers being modern and new to them; (c) gaining a useful generally applicable skill; and (d) a skill taught in an easy-to-understand way. Some suggested that CIRCuiTS challenged their preconception that using the computer is difficult. The two participants who did not like the therapy being on a computer said this was because they found it difficult to grasp (see Table 4).

### Design

All participants spoke positively about the design of the programme, particularly the layout, the virtual village and that tasks generally take place in the “school”, although some would have preferred to have accessed some tasks through different buildings for variation. Several participants expressed an annoyance at the repetitiveness of the music and were unaware that it could be muted.

### Tasks

Participants differed on their favourite and least enjoyable tasks. Reasons for not enjoying particular tasks included finding them difficult to understand or to get completely right, which sometimes led to a sense of failure, despite a good score. Participants cited enjoyment at improving on tasks.

### Perceived cognitive changes

Many participants cited perceived memory improvements. To a lesser extent, participants said that CIRCuiTS had helped their attention and concentration, or their problem solving or planning. Some said that the therapy alone was not enough to change their general thinking skills, but mentioned other benefits such as improving their confidence which had, in turn, allowed them to function better in everyday situations. Others felt that it had done neither, but had simply been enjoyable, fun, or provided a routine.

### Transfer: real life application of CR principles

The majority of participants said they had applied strategies learnt during the therapy to real life situations (all were asked directly about this, and prompted to provide an example). The most common application was of memory aids (e.g. making a list or keeping diaries). Others included attentional strategies in tasks such as writing job applications or following recipes. Three participants said they did not use strategies in real life situations because they felt that what they had learnt during therapy was not transferable. One felt they preferred to use their own methods.

### The therapist

Participants were unanimous in liking their therapist. The majority mentioned personal qualities of their therapist such as friendliness, politeness, patience or positivity. Other participants referred to beneficial qualities of the therapeutic relationship, which was described as trusting, understanding and validating. Most mentioned that the therapists were good teachers who explained things clearly, and six participants mentioned ways in which the therapists had helped them with other aspects of their life, such as signposting them to activities they might be interested in.

### Endings

With regard to the end of each session, 16 participants felt an improvement relative to the start of that session. The majority of participants felt a sense of motivation, confidence, reflection or achievement directly after the session. Exceptions were one participant who said they felt “a bit frustrated” at times and another who felt indifferent.

With regard to the end of the complete course of therapy, responses included (a) relief that they would have more free time; (b) a sense of achievement that they had completed the course; and (c) sadness either because they would miss the therapist or the enjoyment of the tasks. Those who felt sad all said that they were able to deal with this emotion.

### Session frequency and length

Thirteen participants were happy with the length of sessions (mean, 45 minutes) and number of sessions (mean, 26 sessions). Some would have preferred shorter, more frequent sessions, and others suggested they would prefer longer sessions less frequently. Three participants said that they would have liked more sessions; none said they would have preferred fewer, although two had dropped out after only one session.

## Discussion

As far as we know, this is the first set of studies to report explicit attempts to gather and make use of information regarding the acceptability to service users and clinicians in the development of a CR programme for people with a diagnosis of schizophrenia. In the first three studies, CIRCuiTS exceeded high a priori targets for acceptability, comprehensibility and ease of use, with very high consistency for non-clinical participants from the general public, patients with a diagnosis of schizophrenia and CR therapists working within the NHS. Where occasional targets were not met, or qualitative suggestions for improvements were made, these were incorporated into the design of the programme through a series of iterative amendments following high consensus from expert therapists and the research team. The fourth, qualitative study explored a number of themes of importance to service users, which not only generally endorsed the acceptability of CIRCuiTS, but may be hypothesis-generating in future studies investigating factors relating to the process and outcomes of therapy, such as the importance of the therapeutic relationship (e.g. Huddy, Reeder, Kontis, Stahl and Wykes, 2012). For example, one service user commented that increased behavioural activation had been a positive side-effect of receiving CIRCuiTS. This might be a fruitful future means of measuring functional changes following CR.

In the fourth study, with some consistency, participants reported that (a) they found receiving the therapy on a computer to be a positive experience, even when they had had little experience of using a computer; (b) they perceived cognitive improvements as a result of receiving CIRCuiTS; (c) they had at least attempted to apply CR strategies to everyday activities (this transfer of new cognitive skills to daily living is an explicit focus of CIRCuiTS); and (d) the ending of therapy was not a major concern for them. In addition, in contrast to the findings of Rose et al. (2008) where low self-esteem had appeared to result for those who felt their cognition did not improve following the therapy, none of the participants in this study reported reduced levels of self-esteem. Rose and colleagues suggested that a suitable solution for those who do not notice improvements might be found in changing therapist delivery. Our findings are consistent with this having happened, as participants reported that their relationship with the therapist was a significant source of self-esteem, and that therapists helped to create an environment in which participants felt comfortable. Alternatively, self-esteem may have been improved as a result of the computer programme, which may now be better at titrating performance and tailoring tasks to be at a more manageable level for each participant. An important outstanding question for CR is to what extent the therapist's role is an important intrinsic non-specific factor that promotes engagement and change. One study demonstrated that service users who rated the working alliance between themselves and their therapist more favourably, stayed in CR therapy longer and were more likely to improve on their main target complaint, but not on working memory performance or self-esteem (Huddy et al., 2012). There is also evidence from studies of internet-based cognitive behaviour therapy that effect sizes are significantly larger when there is therapist support (see Spek et al., 2007 for a meta-analysis).

The studies we report here provide an exemplar of how to use a mixed method approach in developing therapies with high acceptability to end users. Such studies are rare but can provide invaluable information relating to the likely future uptake and durability of a programme. For example, Drake et al. (2013), in a mixed-method study of the feasibility and acceptability of an online mood-tracking and feedback tool, identified that whilst it appeared to be a valid measure of mood, a number of key adjustments would need to be made to ensure participants were sufficiently motivated to use it on an ongoing basis. Such a study presumably provides a significant short-cut to discovering the feasibility and acceptability of the programme, without which time-consuming pilot studies may have been conducted that were likely to be hampered by consumer dissatisfaction. Rigorous acceptability testing may be particularly important as e-health plays an increasingly important role in the development of new assessments and interventions in mental health, and the design of these programmes tends to be led by researchers and clinicians rather than e-learning experts. Attempts have been made to ensure that internet-based programmes conform to industry quality standards but these are rare (e.g. Rotondi et al., 2012). Furthermore, it may not be appropriate to conform to standard design models, which may have emerged within other areas such as physical health, and thus may need to be adapted for people with mental health problems (Brunette et al., 2012).

One of the potential pitfalls of gathering service users' views is that outcome measures are usually designed by clinicians and researchers, who may have a different view to service users on the most important factors for therapy success. Furthermore, research has shown that therapy evaluations tend to be biased positively because service users are more likely to report high rates of satisfaction when they are interviewed by someone with a vested interest in the therapy (Rose, Wykes, Leese, Bindman and Fleischmann, 2003). Whilst these criticisms may be directed at our first three studies, attempts to minimize the potential for bias were made by: (a) adhering to very basic questions regarding attractiveness, comprehensibility and ease of use; and (b) encouraging, all participants to also report bugs or errors in the programme to improve the design quality, thus priming the expectation that negative criticisms were invited. The fact that the ratings made by service users, therapist and non-clinical users (who presumably had very diverse sets of expectations) exceeded targets perhaps argues against the possibility that poor expectations of service users can lead to heightened satisfaction reports (Williams, Coyle and Healy, 1998).

The fourth study explicitly aimed to address the two pitfalls described relating to service evaluation as well as adopting qualitative methods to try to elicit full responses. The participatory method aims to maximize the potential for criticism of the therapy and minimize coercion of positive views or attitudes towards the therapy with meaningful measures. Furthermore, this study was kept independent from the feasibility randomized controlled trial from which the participants were drawn. Despite these factors, feedback proved to be predominantly positive on all topics covered in the interviews.

In summary, to maximize the longevity and uptake of psychological therapy programmes (particularly e-health programmes) within health services, careful testing of the feasibility and acceptability to service users and clinician users should be undertaken and the results used to improve therapy designs. Commercially available CR programs should be evaluated in terms of acceptability and feasibility and whilst this information is largely neglected, many of these programmes should be considered cautiously. CIRCuiTS is a computerized CR programme designed for people with psychosis, which is based on a clear metacognitive theory of the relationship between cognitive and functional change, has an internal focus on transfer of new cognitive skills to daily life, and is highly flexible in tailoring to individual patients. It may be unique in demonstrating high acceptability and ease of use for service users with a schizophrenia diagnosis and clinicians and having been developed with significant user consultation.

## Supplementary material

For supplementary material accompanying this paper visit http://dx.doi.org/10.1017/S1352465815000168. click here to view supplementary material
