# Supplementary material for: The Feasibility and Acceptability to Service Users of CIRCuiTS, a Computerized Cognitive Remediation Therapy Programme for Schizophrenia
Source: Behav Cogn Psychother. 2015 May 25;44(3):288–305. doi: 10.1017/S1352465815000168 (PMC4855993; doi:10.1017/S1352465815000168)
Supplement: Supplementary file 1 [file S1352465815000168sup.zip › S1352465815000168sup001.doc]

**Appendix 1: Self-assessment measure (SAM)**

For each item, please circle the number you feel best describes your experience.

Thinking about how attractive the software is, how would you rate the following?

|  | Very unattractive |  |  |  | Very attractive   |
| --- | --- | --- | --- | --- | --- |
| Overall programme | 1 | 2 | 3 | 4 | 5 |
| Introduction | 1 | 2 | 3 | 4 | 5 |
| CiRCuITS village | 1 | 2 | 3 | 4 | 5 |
| CiRCuITS music | 1 | 2 | 3 | 4 | 5 |
| Instruction page | 1 | 2 | 3 | 4 | 5 |
| Demos | 1 | 2 | 3 | 4 | 5 |
| Final scoring and rating page | 1 | 2 | 3 | 4 | 5 |
| Library scene | 1 | 2 | 3 | 4 | 5 |

Thinking about how easy the programme is to understand, how would you rate the following?

|  | Very difficult to understand |  |  |  | Very easy to understand   |
| --- | --- | --- | --- | --- | --- |
| Overall | 1 | 2 | 3 | 4 | 5 |
| Task Instructions | 1 | 2 | 3 | 4 | 5 |
| Demos | 1 | 2 | 3 | 4 | 5 |
| Task difficulty/length of task box | 1 | 2 | 3 | 4 | 5 |
| Strategy selector box | 1 | 2 | 3 | 4 | 5 |
| Books in the Library | 1 | 2 | 3 | 4 | 5 |

Thinking about how user-friendly the programme is, how would you rate the following?

|  | Very difficult to use |  |  |  | Very user-friendly   |
| --- | --- | --- | --- | --- | --- |
| Overall | 1 | 2 | 3 | 4 | 5 |
| Navigating the Village | 1 | 2 | 3 | 4 | 5 |
| Instruction page | 1 | 2 | 3 | 4 | 5 |
| Demos | 1 | 2 | 3 | 4 | 5 |
| Task difficulty/length of task box | 1 | 2 | 3 | 4 | 5 |
| Strategy selector box | 1 | 2 | 3 | 4 | 5 |
| Final scoring and rating page | 1 | 2 | 3 | 4 | 5 |
| Books in the Library | 1 | 2 | 3 | 4 | 5 |

| In general, did you find the CIRCUITS programme culturally acceptable? | | | | |
| --- | --- | --- | --- | --- |
| Very unacceptable |  |  |  | Very acceptable   |
| 1 | 2 | 3 | 4 | 5 |

If you were not happy with any aspect of the CIRCUITS programme, please could you explain why.

____________________________________________________________________________________________________________________________________________________________________________________________________________________

Please make any additional comments below.

______________________________________________________________________________________________________________________________________________________________________________________________________________________________________________________________________________________________________________________________

*Thank you for completing this questionnaire, we really appreciate your help.*
