# Supplementary material for: The Feasibility and Acceptability to Service Users of CIRCuiTS, a Computerized Cognitive Remediation Therapy Programme for Schizophrenia
Source: Behav Cogn Psychother. 2015 May 25;44(3):288–305. doi: 10.1017/S1352465815000168 (PMC4855993; doi:10.1017/S1352465815000168)
Supplement: Supplementary file 1 [file S1352465815000168sup.zip › S1352465815000168sup002.doc]

**Appendix 2: Therapist Assessment Measure (TAM)**

For each item, please circle the number you feel best describes your experience of using the feature of CIRCUITS that is described.

1. Firstly, we would like to ask you about some of the features of the software available when completing the tasks.

a) Features *before* beginning a task:

**Thinking about how attractive the software is, how would you rate the following?**

|  | Very unattractive |  |  |  | Very attractive |
| --- | --- | --- | --- | --- | --- |
| Instruction Page | 1 | 2 | 3 | 4 | 5 |
| Demos | 1 | 2 | 3 | 4 | 5 |
| Strategies | 1 | 2 | 3 | 4 | 5 |

**Thinking about how easy the programme is to understand, how would you rate the following?**

|  | Very difficult to understand |  |  |  | Very easy to understand |
| --- | --- | --- | --- | --- | --- |
| Instruction Page | 1 | 2 | 3 | 4 | 5 |
| Demos | 1 | 2 | 3 | 4 | 5 |
| Strategies | 1 | 2 | 3 | 4 | 5 |
| Difficulty and task length ratings | 1 | 2 | 3 | 4 | 5 |

**Do you find the following features helpful when using CiRCuITS with a client?**

|  | Very unhelpful |  |  |  | Very helpful |
| --- | --- | --- | --- | --- | --- |
| Demos | 1 | 2 | 3 | 4 | 5 |
| Strategy demos | 1 | 2 | 3 | 4 | 5 |
| Difficulty and task length ratings | 1 | 2 | 3 | 4 | 5 |

**Thinking about the amount information provided before beginning the task, how would you rate the following?**

| Too much/too little (please circle) |  |  |  | Right amount |
| --- | --- | --- | --- | --- |
| 1 | 2 | 3 | 4 | 5 |

**Features *during* a task:**

Do you find the following features helpful when using CiRCuITS with a client?

|  | Very unhelpful |  |  |  | Very helpful |
| --- | --- | --- | --- | --- | --- |
| ‘i’ (instruction) button | 1 | 2 | 3 | 4 | 5 |
| ‘?’ (hint) function | 1 | 2 | 3 | 4 | 5 |
| ‘s’ (strategy) button | 1 | 2 | 3 | 4 | 5 |
| ‘T’ (therapist – pause) button | 1 | 2 | 3 | 4 | 5 |
| Therapist redo button | 1 | 2 | 3 | 4 | 5 |
| Therapist draw button | 1 | 2 | 3 | 4 | 5 |
| Audio feedback | 1 | 2 | 3 | 4 | 5 |
| Visual feedback | 1 | 2 | 3 | 4 | 5 |

**How would you rate the amount of information provided by the ‘i’ (instruction)?**

| Too much/too little (please circle) |  |  |  | Right amount |
| --- | --- | --- | --- | --- |
| 1 | 2 | 3 | 4 | 5 |

**Would you say that the ‘?’ (hint) function appears at appropriate or inappropriate times?**

| At inappropriate times |  |  |  | At appropriate times |
| --- | --- | --- | --- | --- |
| 1 | 2 | 3 | 4 | 5 |

**Features *after* a task:**

**Thinking about how attractive the software is, how would you rate the following?**

|  | Very unattractive |  |  |  | Very attractive |
| --- | --- | --- | --- | --- | --- |
| Final Rating Page | 1 | 2 | 3 | 4 | 5 |
| Library Books | 1 | 2 | 3 | 4 | 5 |

**Thinking about how easy the programme is to understand, how would you rate the following?**

|  | Very difficult to understand |  |  |  | Very easy to understand |
| --- | --- | --- | --- | --- | --- |
| Score bar | 1 | 2 | 3 | 4 | 5 |
| Library Help book | 1 | 2 | 3 | 4 | 5 |

**Do you find the following features helpful when using CiRCuITS with a client?**

|  | Very unhelpful |  |  |  | Very helpful |
| --- | --- | --- | --- | --- | --- |
| Score bar | 1 | 2 | 3 | 4 | 5 |
| Difficulty ratings | 1 | 2 | 3 | 4 | 5 |
| Time taken feedback | 1 | 2 | 3 | 4 | 5 |
| Strategy rating | 1 | 2 | 3 | 4 | 5 |
| Library Score book | 1 | 2 | 3 | 4 | 5 |
| Library Help book | 1 | 2 | 3 | 4 | 5 |

2. Secondly, we would like to ask you about how you find some of the therapist-only features.

**Thinking about how easy to use the software is, how would you rate the following?**

|  | Difficult |  |  |  | Easy |
| --- | --- | --- | --- | --- | --- |
| Using client view | 1 | 2 | 3 | 4 | 5 |
| Skip and reset buttons | 1 | 2 | 3 | 4 | 5 |
| Therapist Manual | 1 | 2 | 3 | 4 | 5 |
| Transfer data to software for audit / research | 1 | 2 | 3 | 4 | 5 |

**Thinking about how attractive the software is, how would you rate the following?**

|  | Very unattractive |  |  |  | Very attractive |
| --- | --- | --- | --- | --- | --- |
| Client view | 1 | 2 | 3 | 4 | 5 |
| Therapist manual | 1 | 2 | 3 | 4 | 5 |

3. Finally, we would like to know what you think about the software overall.

How would you rate the following?

| **Attractiveness?** | | | | |
| --- | --- | --- | --- | --- |
| Unattractive |  |  |  | Attractive |
| 1 | 2 | 3 | 4 | 5 |

| **Ease of use?** |  |  |  |  |
| --- | --- | --- | --- | --- |
| Difficult to use |  |  |  | Easy to use |
| 1 | 2 | 3 | 4 | 5 |

| **Ability to understand?** | | | | |
| --- | --- | --- | --- | --- |
| Difficult to understand |  |  |  | Easy to understand |
| 1 | 2 | 3 | 4 | 5 |

If you were not happy with any aspect of the CIRCUITS programme, please could you explain why.

____________________________________________________________________________________________________________________________________________________________________________________________________________________

Please make any additional comments below.

______________________________________________________________________________________________________________________________________________________________________________________________________________________________________________________________________________________________________________________________

*Thank you for completing this questionnaire, we really appreciate your help.*
