# Supplementary material for: The Feasibility and Acceptability to Service Users of CIRCuiTS, a Computerized Cognitive Remediation Therapy Programme for Schizophrenia
Source: Behav Cogn Psychother. 2015 May 25;44(3):288–305. doi: 10.1017/S1352465815000168 (PMC4855993; doi:10.1017/S1352465815000168)
Supplement: Supplementary file 1 [file S1352465815000168sup.zip › S1352465815000168sup003.doc]

**Appendix 3: Therapist Semi-Structured Interview (TSSI)**

1. Now that you have created therapy packages on CiRCuITS for five hypothetical clients, what do you think about this function of the programme?
2. How useful do you think this feature is and can you see yourself using it for your clients?
3. Given what you know about the tasks in CiRCuITs, do you think you will be able to tailor the programme to your clients? How easy or difficult do you envisage this being?
4. Is there anything you can think of which would help you to deliver computerised CRT to your clients?
5. Have you got any ideas about how to improve CiRCuITS generally?

Possible prompt/steering questions:

- Can you tell me a little bit more?
- Is there anything else that you can tell me?
- Can you be more specific?
- How easy/difficult do you find that?
